# The Emerging Roles of Pericytes in Modulating Tumor Microenvironment

**DOI:** 10.3389/fcell.2021.676342

**Published:** 2021-06-11

**Authors:** Ruipu Sun, Xiangzhan Kong, Xiaoyi Qiu, Cheng Huang, Ping-Pui Wong

**Affiliations:** ^1^Guangdong Provincial Key Laboratory of Malignant Tumor Epigenetics and Gene Regulation, Guangdong-Hong Kong Joint Laboratory for RNA Medicine, Sun Yat-sen Memorial Hospital, Sun Yat-sen University, Guangzhou, China; ^2^Medical Research Center, Sun Yat-sen Memorial Hospital, Sun Yat-sen University, Guangzhou, China

**Keywords:** pericyte, mural cell, tumor microenvironment, angiogenesis, immunomodulation

## Abstract

Pericytes (PCs), known as mural cells, play an important blood vessel (BV) supporting role in regulating vascular stabilization, permeability and blood flow in microcirculation as well as blood brain barrier. In carcinogenesis, defective interaction between PCs and endothelial cells (ECs) contributes to the formation of leaky, chaotic and dysfunctional vasculature in tumors. However, recent works from other laboratories and our own demonstrate that the direct interaction between PCs and other stromal cells/cancer cells can modulate tumor microenvironment (TME) to favor cancer growth and progression, independent of its BV supporting role. Furthermore, accumulating evidence suggests that PCs have an immunomodulatory role. In the current review, we focus on recent advancement in understanding PC’s regulatory role in the TME by communicating with ECs, immune cells, and tumor cells, and discuss how we can target PC’s functions to re-model TME for an improved cancer treatment strategy.

## Introduction

Pericytes (PCs) are embedded in the basement membrane of blood microvessels (Bergers and Song, 2005), which play a vital role in regulating physiological and pathological events, including vascular development, homeostasis, fibrosis, and stroke. Generally, PCs are responsible for the regulation of vascular stabilization, vascular permeability, blood flow, and angiogenesis along with endothelial cells (ECs) in blood vessels (BVs) (Hellstrom et al., 2001; Pallone and Silldorff, 2001; Enge et al., 2002). In angiogenesis, sprouting ECs secrete platelet derived growth factor (PDGF) to recruit platelet derived growth factor receptor-beta (PDGFR-β) positive mural cells (including PCs), which then interact with ECs and stabilize the newly formed BVs (Carmeliet and Jain, 2000). Unlike other stromal cells, PCs can be distinguished by dynamic molecular marker expression pattern under different conditions, such as PDGFR-β, CD13 (alanine aminopeptidase), Cluster of differentiation 146 (CD146), alpha-smooth muscle actin (α-SMA) (Dermietzel and Krause, 1991; Lindahl et al., 1997; Ozerdem et al., 2001). In recent years, PCs are defined as heterogeneous, tissue-specific, and multipotent cell population in BVs (Ferland-McCollough et al., 2017), which are mainly due to their tissue/organ-specific roles (Shepro and Morel, 1993; Armulik et al., 2005; Corselli et al., 2013; Kitano and Bloomston, 2016) and ability to give rise to various cell populations (Dore-Duffy et al., 2006; Dellavalle et al., 2007; Crisan et al., 2008; Olson and Soriano, 2011). During tumor angiogenesis, defective EC–PC interaction is one of the major causes of the formation of dysfunctional tumor vasculature and hypoxic tumor microenvironment (TME), which favors cancer growth and metastasis (Song et al., 2005). Therefore, it is important to investigate the underlying role of PCs in modulating tumor angiogenesis and TME in order to develop an improved anti-cancer treatment.

Anti-angiogenetic therapy is recognized as a promising treatment strategy for cancer, while many anti-angiogenic drugs have been approved for certain types of cancers, including anti-vascular endothelial growth factor (VEGF) drug (i.e., Bevacizumab, Ranibizumab) and some tyrosine kinase inhibitors (i.e., Sorafenib, Sunitinib) (Meng et al., 2015; Ramjiawan et al., 2017; Li et al., 2018). However, the resistance to anti-angiogenetic therapy have jeopardized their clinical benefits in cancer patients (Ramjiawan et al., 2017; Li et al., 2018). Previous studies suggested that PCs can protect ECs from anti-angiogenic therapies probably by secreting pro-angiogenic factors (Franco et al., 2011) or soluble factors (Prete et al., 2018). In addition, PCs may increase their coverage around tumor BVs adaptively and cause resistance to anti-angiogenetic therapy in preclinical models (Benjamin et al., 1999; Bergers and Hanahan, 2008). Nevertheless, combination treatment with PDGF receptor kinase inhibitor erlotinib/imatinib and bevacizumab showed very limited benefits in the clinical trials and even displayed an additive toxicity in some cancer patients (Hainsworth et al., 2007). The failure behind these trials suggests that PC may have other potential roles in controlling tumor growth and progression. Indeed, recent work from our laboratory shows that PC can regulate tumor cell growth via paracrine signals controlled by β3-integrin (Wong et al., 2020), independent of its BV supporting function, suggesting that its regulatory role in the TME is far more complicated than we previously thought.

In this review, we will exploit the current progress of understanding the role of PC in regulating TME, and discuss its functions in regulating tumor cells and other stromal cells to dictate cancer growth and progression. For comprehensive reviews of its role in BV formation and supporting function, please see Betsholtz and Crivellato (Armulik et al., 2011; Ribatti et al., 2011).

## Crosstalk Between Pericytes and Tumor/Stromal Cells in Tumor Microenvironment

Although the composition of TME varies in different cancer types, some features seem to be typical characteristics in most solid tumors. Indeed, TME consists of cancer cells and some non-malignant cells, including ECs, PCs, immune inflammatory cells, cancer-associated fibroblasts (CAFs), and also extracellular matrix (ECM) components (including cytokines, chemokines, matrix metalloproteinases, integrins, and other secreted molecules) (Hanahan and Weinberg, 2011). In this section, we review and discuss the multifaceted roles of PCs in regulating tumor cell and stromal cell’s functions in details.

### Abnormal Endothelial Cell–Pericyte Interaction and Signaling in Tumor Vasculature

Endothelial cells are the fundamental cells lining the interior face of BV walls, which are surrounded by quiescent mural cells (including PCs). PCs are capable of interacting with newly proliferating ECs to form nascent BVs and secrete angiogenetic factors to stabilize the newly-formed vessels (Abramsson et al., 2003). In tumorigenesis, defective EC–PC interaction leads to the formation of disorganized tumor vasculature (Ferland-McCollough et al., 2017). This is because PC is an essential mediator to maintain the integrity of tumor BVs, while PDGF-B/PDGFR-β signaling is critical for controlling PC migration during angiogenesis. Preceding findings have suggested that PDGFR-β mediated paracrine loop activates ECs to produce PDGF-B in order to recruit PDGFR-β-positive PCs, which in return releases VEGF-A and Ang-1 to stabilize the newly formed BVs (Armulik et al., 2005). Afterward, Ang-1 regulates the maturation and integrity of BV through binding to the endothelial cell-Tie-2 receptor (Harrell et al., 2018). During sprouting angiogenesis, ECs can also secrete Ang-2 to compete with Ang-1 for the binding to endothelial cell-Tie-2 receptor, which in turn destructs EC–PC interaction and destabilizes BVs (Saharinen et al., 2017). Interestingly, overexpression of PDGF-B by ECs causes an increase in PC coverage and vascular stability as well as accelerated tumor progression (Guo et al., 2003; Furuhashi et al., 2004). Moreover, tumor-derived PDGF-B induces endothelial cell-SDF-1α secretion, which then promotes PC migration and recruitment during tumor angiogenesis (Song et al., 2009). Furthermore, EC- and PC-derived HB-EGF (heparin-binding epidermal growth factor-like growth factor) activates EGFR (epidermal growth factor receptor) specifically in tumor-associated perivascular cells, resulting in increased PC coverage and enhanced angiogenesis (Nolan-Stevaux et al., 2010). Conversely, it has been suggested that inadequate PDGF-B in the stroma results in inappropriate attachment of PCs to ECs (Raza et al., 2010). Previous works have demonstrated that the blockade of Notch signaling inhibits vascular co-option and disrupts the EC-PC interaction during tumor angiogenesis (Hernandez et al., 2013), while Jagged-1 expression and Notch signaling are shown to be important for the growth of ECs and PCs as well as the maintenance of EC–PC interaction (Tattersall et al., 2016). In the study of Meng et al. (2015), they discover that ECs and PCs can build an “EC-PC shield” to protect tumor cells from cancer-directed therapy and immune surveillance in the TME, while the maintenance of BV integrity ensures an adequate oxygen and nutrient supply to tumor cells, which in turn promotes cancer growth and progression (Ferland-McCollough et al., 2017). Indeed, clinical studies show that high BV’s PC coverage is associated with increased tumor growth and poor prognosis (Furuhashi et al., 2004), while it is correlated with reduced distant metastasis in colorectal cancer patients (Yonenaga et al., 2005). Overall, these findings suggest that PC overabundance and deficiency occur in different tumor types during vascularization with mixed clinical outcome, implying that targeting PC coverage may not be an ideal strategy for anti-cancer treatment. Instead, our recent study indicates that PC-derived paracrine signals can modulate tumor cell growth independent of its BV supporting role and coverage (Wong et al., 2020), suggesting that targeting PC derived paracrine signals could be an alternative method for cancer therapy.

### Direct Paracrine Crosstalk Between Tumor Cells and Pericytes Determines Cancer Growth and Progression

Although PCs have been considered as a critical compartment of the TME, the underlying mechanism of tumor cell–PC interaction has yet to be elucidated. Recently, we have shown that high percentage of mural-β3-integrin negative BVs correlates with increased tumor size and progression in multiple cancers (Wong et al., 2020), while PC specific knock out of β3-integrin expression enhances tumor growth independent of its BV supporting role. Further mechanistic study shows that loss of PC-β3-integrin expression increases the production of paracrine factors, including CCL2, CXCL1, and TIMP1, via activation of the FAK-HGFR-Akt-NF-κB signaling pathway, while PC-derived CCL2 enhances MEK1-ERK1/2-ROCK2 mediated tumor growth, suggesting that inhibition of ROCK in tumors with low PC-β3-integrin expression could potentially control cancer growth (Wong et al., 2020). Interestingly, a recent study from Lechertier et al. (2020) show that loss of PC FAK enhances p-Pyk2-Gas6-Axl-Akt signaling and its downstream Cyr61 expression to stimulate tumor angiogenesis, while PC-derived Cyr61 is also able to enhance tissue factor expression in tumor cells and its mediated cell proliferation. This work provides first evidence that PCs can crosstalk with ECs and tumor cells via the same paracrine signal (Lechertier et al., 2020). Furthermore, Caspani et al. study shows that a pathogenic crosstalk between PCs and tumor cells determines glioblastoma progression in mouse models (Caspani et al., 2014).

### Pericytes Modulate Immunosuppressive Tumor Microenvironment

**FIGURE 1:**
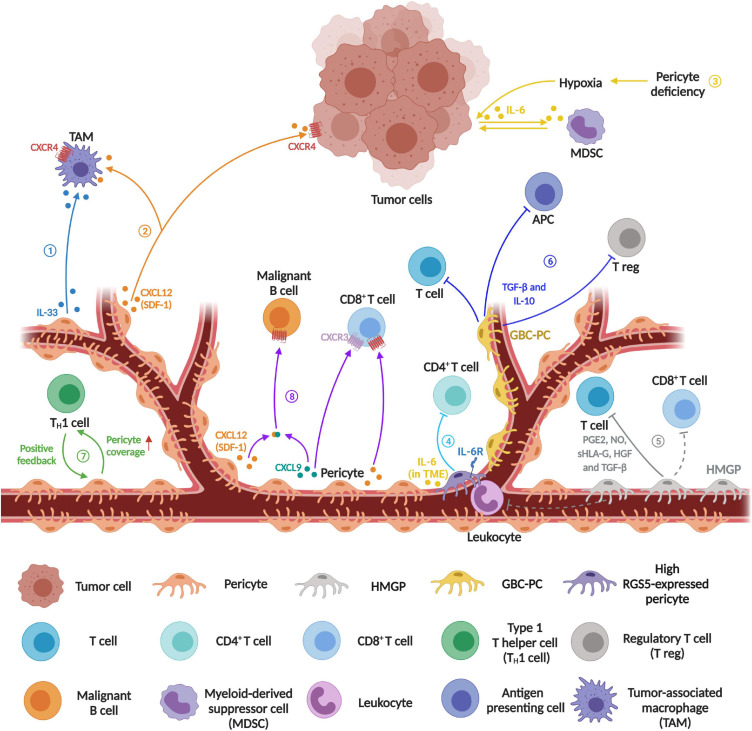
Schematics diagram represents the emerging immunomodulatory role of pericytes in tumor microenvironment. ➀Recruitment of tumor-associated macrophage (TAM). PDGF-BB-stimulated PCs release IL-33 to recruit more TAMs. ➁ Increased co-migration of TAMs and tumor cells. PC-derived chemokine CXCL12 (SDF-1) contributes to the co-migration of TAMs and tumor cells during innate immune response. ➂ Increased myeloid-derived suppressor cells (MDSCs) transmigration. PC loss causes leaky blood vessels and inadequate oxygen supply leading to tumor hypoxia, which then induces IL-6 expression in tumor cells to increase MDSC transmigration, resulting in suppression of the T cell-mediated anti-tumor response. ➃ Induced CD4^+^ T cell anergy. Tumor PCs act as negative regulators of CD4^+^ T cell activity. ➄ Inhibition of mitogen- and allogeneic-stimulated T cell proliferation. Human malignant glioma-derived pericyte (HMGP) releases PGE2, NO, sHLA-G, HGF, and TDF-β to suppress T cell proliferation, while CD90-positive PCs may function as suppressors of the infiltration of leukocytes and CD8^+^ T cells in malignant glioma. ➅ Inhibition of T cell and antigen presenting cell activity, and increased recruitment of regulatory T cells. Glioblastoma conditioned-pericyte (GBC-PC) not only negatively regulates T cell and antigen presenting cell (APC) but also recruits regulatory T cell (T reg). ➆ Regulation of blood vessel normalization and immune cell infiltration. In the positive feedback loop between type 1 T helper (T_H_1) and blood vessel normalization, PC coverage has a certain impact on T_H_1-mediated immune cell infiltration. ➇ Enhanced CD8^+^ T cell recruitment and malignant B cell migration. Perivascular cell derived CXCL9 and CXCL12 can recruit CD8^+^ T cell effectors by binding to their corresponding receptor CXCR3 and CXCR4 respectively. Besides, CXCL9 forms a heterocomplex with CXCL12, which then enhances CXCR4-dependent malignant B cell migration to accumulate on the vessel wall (Created with BioRender.com).

Inflammatory cells, an important component in the TME, are often associated with the inflammatory and immune responses in carcinogenesis. It is known that solid tumors are infiltrated by various innate and adaptive immune cells with both pro-tumor and anti-tumor functions (Turley et al., 2015). Previous works have shown that PCs release chemokines and cytokines in response to the pro-inflammatory stimulus, such as CCL2, CCL3, CXCL1, IFN-γ, and IL-8 (Navarro et al., 2016), while they also express some functional pattern-recognition receptors (i.e., TLR4, TLR2, NOD1) and macrophage markers (i.e., ED-2), implying that they may also have a role in modulating immune response (Navarro et al., 2016). Interestingly, PCs display phagocytic and pinocytic ability and regulate different types of leukocytes trafficking (Navarro et al., 2016). Accordingly, tumor PCs have distinct effects on tumor-associated macrophages (TAMs) in TME, while IL-33 produced by PDGF-BB-stimulated PCs has been shown to recruit TAMs in order to promote cancer metastasis in several human and mouse xenograft models (Figure 1, process ➀) (Yang et al., 2016). PC-derived chemokine CXCL12 (SDF-1) can trigger the EGF/CSF-1 paracrine invasion loop to mediate the co-migration of TAM and tumor cells, after binding to its receptor CXCR4 on both TAMs and tumor cells (Figure 1, process ➁) (Qian and Pollard, 2010). Meanwhile, crosstalk between M2-like macrophages and PCs in glioblastoma (GBM) promotes PC recruitment and upregulates the expression of the proangiogenic ECM component periostin deposition in PCs through the CECR1–PDGF-B–PDGFR-β signaling pathway (Zhu et al., 2017). In the *pdgfb*^ret/ret^ mouse model, PCs deficiency-driven hypoxia result in IL-6 upregulation and an increased myeloid-derived suppressor cell (MDSC) transmigration in tumors, and the MDSC accumulation leads to increased tumor growth, while the amounts of circulating malignant cells can be abrogated upon the recovery of PC coverage (Figure 1, process ➂) (Hong et al., 2015).

Newer evidence suggests that tumor-derived PCs regulate the activity and proliferation of T lymphocytes in TME (Figure 1). In a mouse spontaneous model of pancreatic cancer (RIP1-Tag5), knocking out *RGS5* (regulator of G-protein signaling 5) gene results in PC maturation, vascular normalization and consequently a marked reduction in tumor hypoxia and vessel leakiness, while these changes enhance immune cell infiltration and extend the survival of tumor bearing mice (Hamzah et al., 2008). Furthermore, PC-RGS5 overexpression has been observed in several types of human tumors including kidney, liver, and head and neck cancers (Furuya et al., 2004; Hamzah et al., 2008). Coincidently, Bose et al. show that the expression of PC-RGS5 is upregulated after co-cultured with tumor-derived supernatant or established subcutaneous tumors (Bose et al., 2013). Tumor derived PCs inhibit CD4^+^ T cell proliferation and activation while promoting CD4^+^ T cell anergy *in vitro*, which is also regulated by RGS5- and IL-6-dependent signaling pathways (Figure 1, process ➃). In addition, the expression of PD-L1 is up-regulated in PCs after co-cultured with tumor fragments (Bose et al., 2013). These results suggest that the combined effect of PC-PD-L1 and RGS5 expression might protect tumor cells from cytotoxic T cells. In a different study, the authors show that human malignant glioma-derived pericytes (HMGP), which co-expressed CD90, CD248, and PDGFR-β, are capable of inhibiting the proliferation of mitogen- and allogeneic-stimulated T cells via the release of prostaglandin E2 (PGE2), serum human leukocyte antigen G (sHLA-G), hepatocyte growth factor (HGF), and transforming growth factor-beta (TGF-β) (Figure 1, process ➄). Clinically, the expression level of CD90 in perivascular cells positively correlates with glioma malignancy, while it is negatively associated with BV-associated leukocytes and CD8^+^ T cell infiltration (Ochs et al., 2013). Recently, Valdor et al. report that GBM-conditioned-pericytes (GBC-PCs) can secrete a high level of anti-inflammatory cytokines and immunosuppressive molecules while reducing their surface co-stimulatory molecule expression, which in turn suppresses CD4^+^ T cell response and IL-2 production *in vitro* (Figure 1, process ➅) (Valdor et al., 2017). Further study shows that GBC-PCs upregulate chaperone-mediated autophagy (CMA) to enhance the expression of anti-inflammatory cytokines TGF-β and IL-10, which then inhibit T cell and antigen presenting cell activity and recruit regulatory T cells (Figure 1, process ➅) (Valdor et al., 2019). Additionally, PCs contribute to the subsequent positive feedback loop of type 1 T helper cells-mediated vessel normalization and immune response (Figure 1, process ➆) (Tian et al., 2017).

Interestingly, Daniel et al., show that PCs may also possess a potential regulatory role of malignant B cell recruitment in primary central nervous system lymphoma (Figure 1, process ➇). Clinically, the localization and density of activated CD8^+^ T cells within tumors is correlated with the expression level of inflammatory chemokine CXC chemokine ligand 9 (CXCL9), which is an agonist of the CXC chemokine receptor 3 (CXCR3), mainly secreted by PCs and perivascular macrophages. In the perivascular TME, CXCL9 can form heterocomplex with B-cell chemoattractant CXCL12 to enhance CXCL12-induced CD8^+^ T cell as well as malignant B cell recruitment toward BV walls in the primary central nervous system lymphomas (Venetz et al., 2010). In addition, our recent work shows that β3-integrin controls the secretion of CCL2, CXCL1, and TIMP1 from PCs via the FAK-HGFR-Akt-NF-κB signaling (Wong et al., 2020), while these cytokines have been linked to immune cell infiltration and activity in TME (Navarro et al., 2016), suggesting that targeting PC-β3-integrin and its downstream signaling pathway can be a potential strategy to modulate immunosuppressive TME.

### The Role of Pericytes-Fibroblast Transition in Tumor Microenvironment

As a fundamental component of the tumor stroma, cancer associated fibroblast (CAFs) have a role in modulating TME and changing the behavior of neoplastic cells in either a tumor-promoting or tumor-inhibiting manner (Kalluri, 2016). In the tumor-promoting property, CAFs support carcinogenesis through secretion of cytokine, growth factors and angiogenic factors, production and remodeling of the ECM, as well as suppression of immune surveillance in the TME (Matsuda and Seki, 2020). Recently, PC is considered to be one of the major sources of CAFs in tumors and fibrosis (Öhlund et al., 2014; Kalluri, 2016). A novel finding reveals that PDGF-BB-PDGFRβ signaling can induce pericytes-fibroblast transition (PFT), while the detached PCs from tumor microvasculature can transdifferentiate to fibroblasts that significantly contributed to tumor invasion and metastasis (Hosaka et al., 2016).

## Targeting Pericytes as a Cancer Treatment Strategy: Challenges vs Opportunities

It has become a research hot topic for developing direct/indirect PC-targeted agents against angiogenesis and cancer growth in the last decades (Supplementary Table 1). However, majority of these agents showed modest or no effect on tumor growth and progression as a single agent in preclinical animal models, especially for PDGFR-targeted therapy. Combining anti-PDGFR agent with chemotherapy or other agent-targeted therapy displayed slightly better anti-tumor effect in mouse models of certain cancer types (Supplementary Table 1). Furthermore, the phase 3 clinical trials of PC-related antitumor therapy have so far shown modest clinical benefits in certain cancers (Table 1). Besides, the combination therapy of anti-PDGFB and anti-VEGF had very limited effect in the clinical trials and even showed additive side effects in some patients (Hainsworth et al., 2007). After interpreting these studies, we speculate that drug dosing strategy is a critical variable which may determine whether PC-targeted drugs promote vascular function and immune cell infiltration or induce tumor vasculature destruction and cancer metastasis. Therefore, it is a clinically unmet need to investigate how to target PC coverage or recondition PC functions (i.e., immunomodulatory role) for preferred immunobiology/vascular function in TME. Apart from targeting PCs directly, Cantelmo et al. show that inhibition of the glycolytic activator PFKFB3 in ECs induces tumor vessel normalization to improve PC coverage and chemotherapy delivery in the preclinical models. The authors also claimed that depletion of PFKFB3 significantly inhibits placenta derived PC proliferation, while improves PC coverage and adherence to ECs in tumor BVs (Cantelmo et al., 2016). However, the short-term effect of BV normalization raises a question about its application in the clinic (Wong et al., 2015). Recently, we discover that loss of mural-β3-integrin expression significantly enhances FAK-p-HGFR-p-Akt-p-p65 mediated CCL2 cytokine production, which in turn activates CCR2-MEK1-ROCK2 dependent tumor growth (Wong et al., 2020). These findings suggest that cancer patients with low PC-β3-integrin expression can be potentially treated with CCR2 or ROCK inhibitors.

**TABLE 1:** Phase 3 clinical trials of Pericyte-related antitumor therapy.

**Cancer type**	**Treatment**	**Targets**	**Results**	**References**
Temozolomide-resistant progressive GBM	Imatinib + hydroxyurea vs hydroxyurea	PDGFR, c-Kit, and BCR-Abl	Imatinib does not improve PFS in combination therapy.	Dresemann et al., 2010
GIST (failure of imatinib and sunitinib treatment)	Imatinib vs placebo		Resumption of imatinib improves PFS and disease control at 12 weeks.	Kang et al., 2013
Unresectable or metastatic GIST	Imatinib vs nilotinib	PDGFR, c-Kit, and BCR-Abl; PDGFR, BCR-Abl, DDR1, and c-Kit	PFS is higher in the imatinib group than in the nilotinib group.	Blay et al., 2015
Radioiodine-refractory thyroid cancer	Lenvatinib vs placebo	PDGFR,VEGFR, FGFR, c-Kit, and Ret	Lenvatinib improves in PFS and the response rate but has more adverse effects.	Schlumberger et al., 2015
Advanced HCC	Sorafenib vs placebo	PDGFR, VEGFR, Raf, and c-Kit	Sorafenib prolongs median survival and time-to-radiologic-progression in patients.	Rimassa and Santoro, 2009
Advanced HCC	Sorafenib vs placebo		Sorafenib improves median OS significantly.	Cheng et al., 2009
HCC	Sorafenib vs placebo		Sorafenib therapy is not efficacious after HCC resection or ablation.	Bruix et al., 2015
Radioiodine-refractory, locally advanced or metastatic differentiated thyroid cancer	Sorafenib vs placebo		Sorafenib significantly improves PFS.	Brose et al., 2014
Non-metastatic RCC	Sorafenib or sunitinib vs placebo	PDGFR, VEGFR, Raf, and c-Kit; PDGFR, VEGFR, c-Kit, Flt3, CSF-1R, and Ret	Sorafenib or sunitinib adjuvant treatment shows no survival benefit relative to placebo.	Haas et al., 2016
Advanced GIST	Sunitinib vs placebo	PDGFR, VEGFR, c-Kit, Flt3, CSF-1R, and Ret	Sunitinib shows significant clinical benefit.	Demetri et al., 2006
PNET	Sunitinib vs placebo		Sunitinib improves PFS and OS.	Raymond et al., 2011
ccRCC	Sunitinib vs placebo		Sunitinib improves the median duration of disease-free survival.	Ravaud et al., 2016
Metastatic RCC	Sunitinib vs interferon α	PDGFR, VEGFR, c-Kit, Flt3, CSF-1R, and Ret	Sunitinib improves PFS and response rates.	Motzer et al., 2007
Advanced RCC	Axitinib vs sorafenib	VEGFR; PDGFR, VEGFR, Raf, and c-Kit	Axitinib results in prolonged PFS.	Rini et al., 2011
Advanced NSCLC	Anlotinib vs placebo	PDGFR, VEGFR, FGFR, c-Kit, and Ret	Prolongs OS and PFS.	Han et al., 2018
Advanced or metastatic RCC	Pazopanib vs placebo	PDGFR, VEGFR, FGFR, and c-Kit	Pazopanib improves PFS and tumor response.	Sternberg et al., 2010
Metastatic non-adipocytic soft-tissue sarcoma (failure of standard chemotherapy)	Pazopanib vs placebo		Pazopanib improves PFS significantly.	van der Graaf et al., 2012
Soft tissue sarcoma	Pazopanib vs placebo		Pazopanib improves PFS significantly.	Kawai et al., 2016
Metastatic CRC	Regorafenib vs placebo	PDGFR, VEGFR, Tie2, FGFR, c-Kit, Ret, and Raf	Regorafenib shows survival benefits.	Grothey et al., 2013
HCC (progressed on sorafenib)	Regorafenib vs placebo		Regorafenib provides survival benefits.	Bruix et al., 2017
Advanced GIST (failure of imatinib and sunitinib)	Regorafenib vs placebo		Regorafenib improves PFS.	Demetri et al., 2013
Advanced ovarian cancer	Carboplatin and paclitaxel + placebo vs carboplatin and paclitaxel + nintedanib	PDGFR, VEGFR, and FGFR	Nintedanib in combination with carboplatin and paclitaxel increases PFS.	du Bois et al., 2016
Recurrent ovarian cancer	Paclitaxel + placebo vs paclitaxel + trebananbib	Ang-1 and Ang-2	Trebananib prolongs PFS in paclitaxel treatment.	Monk et al., 2014
Recurrent partially platinum-sensitive/resistant ovarian cancer	Pegylated liposomal doxorubicin + placebo vs Pegylated liposomal doxorubicin + trebananbib		Trebananbib improves ORR and DOR but does not improve the PFS.	Marth et al., 2017
Advanced ovarian cancer	Carboplatin and paclitaxel + placebo vs carboplatin and paclitaxel + trebananbib		Trebananbib does not improve PFS.	Vergote et al., 2019

*ccRCC, clear cell renal cell carcinoma; CRC, colorectal cancer; CSF-1R, colony-stimulating factor 1; DDR1, discoidin domain receptor 1; DOR, duration of response; FGFR, fibroblast growth factor receptor; GBM, glioblastoma; GIST, gastrointestinal stromal tumor; HCC, hepatocellular carcinoma; NSCLC, non-small-cell lung cancer; ORR, objective response rate; OS, overall survival; PDGFR, platelet-derived growth factor receptor; PFS, progression-free survival; PNET, pancreatic neuroendocrine tumor; RCC, renal cell carcinoma.*

## Conclusion

As an obligatory constituent of the TME, PCs modulate the TME by interacting with tumor cells, ECs, immune cells, and CAFs, beyond their BV supporting role. Recent work supports direct cross-talk between PCs and tumor cells in the TME, which can promote tumor growth independent of tumor angiogenesis. Also, the interplay between ECs and PCs in regulating vascular formation and remodeling has been demonstrated in numerous studies. Disrupting EC-PC interactions in tumor vasculature not only affects PC coverage on tumor BVs but also alter vascular and perivascular TME to influence the efficacy of anti-tumor therapies. Indeed, new studies have highlighted that PCs protect tumor cells from immune surveillance through suppressing the proliferation or response of inflammatory cells around the tumor parenchyma, which could be a new potential target for cancer immunotherapy. Besides, the observation of PC-fibroblast transition suggests the potential progenitor cell property of PC in the TME. In this review, we provide new information to support an integral role for PCs in promoting tumor progression in part through their regulatory activities of tumor cells and dominated stromal cells, suggesting that PCs can serve as a therapeutic target for anticancer treatment in addition to anti-angiogenesis. Meanwhile, the stromal cells within TME may also provide potential therapeutic targets for intending anti-angiogenesis combination therapy since their underlying relationships with PCs. Future studies should focus on exploring the underlying mechanisms of PC-stromal cell/tumor cell interaction in the TME in order to identify new therapeutic targets for an improved cancer treatment strategy.

## Author Contributions

RS wrote the manuscript and made the figure as well as the table. XK, XQ, and CH reviewed the manuscript. P-PW conceptualized, wrote, and reviewed the final version. All authors approved the submission for publication.

## Conflict of Interest

The authors declare that the research was conducted in the absence of any commercial or financial relationships that could be construed as a potential conflict of interest.

## Supplementary Material

Supplementary Table 1 Preclinical testing of Pericyte-targeted antitumor therapy.
