# Supplementary material for: The Emerging Roles of Pericytes in Modulating Tumor Microenvironment
Source: Front Cell Dev Biol. 2021 Jun 11;9:676342. doi: 10.3389/fcell.2021.676342 (PMC8232225; doi:10.3389/fcell.2021.676342)
Supplement: Supplementary Table 1 — Preclinical testing of Pericyte-targeted antitumor therapy. [file Table_1.docx]

| **Supplementary Table 1. Preclinical testing of Pericyte-targeted antitumor therapy** | | | | | |
| --- | --- | --- | --- | --- | --- |
| ***Direct pericyte-targeted antitumor therapy (via pericyte markers or pericyte-derived agents)*** | | | | | |
| **Cancer type** | **Treatment** | **Targets** | **Impact on tumor PCs or vasculature** | **Impact on tumor growth** | **Reference** |
| RIP1-Tag2 transgenic mouse model of pancreatic neuroendocrine cancer | Imatinib; SU10944; CTX | PDGFR, c-Kit, and BCR-Abl; VEGFR | Dissociated PC and reduced PC coverage | No effect on the growth of tumors in monotherapy; enhanced anti-tumor efficacy in combination with metronomic chemotherapy or VEGFR inhibition | (Pietras and Hanahan, 2005) |
| CRC in subcutaneous mouse model | Imatinib; bevacizumab | PDGFR, c-Kit, and BCR-Abl; VEGFR | Combination of imatinib with low-dose bevacizumab increases PC coverage and promotes vascular normalization; monotherapy has no effect on PC coverage | Combination therapy inhibits tumor growth; tumor growth is not significantly affected after imatinib monotherapy | (Schiffmann et al., 2017) |
| Lewis lung carcinoma and fibrosarcoma implanted in murine models | PDGFR ectodomain (Ad sPDGFR); VEGFR2/Flk1 ectodomain (Ad Flk1-Fc) | PDGFRβ; VEGFR | Ad sPDGFR blocks PC recruitment and reduces PC coverage; Ad Flk1-Fc decrease PC content | Monotherapy is less effective on tumor than combination therapy | (Kuhnert et al., 2008) |
| RIP1-Tag2 transgenic mouse model of pancreatic islet cancer | SU6668; SU5416;  STI571 | PDGFR; PDGFR, c-Kit, and BCR-Abl; VEGFR | Increased apoptosis and regressed BV in SU6668 and SU5416 combination therapy; reduced abundance and detachment of PC as well as decreased vascularity in SU5416 and STI571 combination therapy | Reduced tumor in SU6668 and SU5416 combination therapy;  combination of STI571 with SU5416 causes late-stage tumor regression | (Bergers et al., 2003) |
| Ovarian carcinoma in orthotropic mouse model | STI571; AEE788; paclitaxel | PDGFR, c-Kit, and BCR-Abl;  VEGFR; | Reduced PC coverage in STI571 group;  decreased PC coverage in the AEE788 plus STI571 group and in the triple combination group | STI571 alone is not effective; triple combination is more effective than all other treatments; greatest improvement in survival and regression of formed tumors in triple combination | (Lu et al., 2007) |
| Human thyroid cancer in subcutaneous mouse model | STI571; PTK/ZK;  taxol | PDGFR, c-Kit, and BCR-Abl; VEGFR; | Decreased PC coverage in monotherapy and combination therapy | No significant effect on tumor growth alone or in combination but the inhibitors enhance the effect of taxol chemotherapy in triple combination | (Kłosowska-Wardega et al., 2009) |
| Human cervical carcinogenesis mouse model | Imatinib | PDGFR, c-Kit, and BCR-Abl | Reduced lesional neovascularization and diminished blood vessel density in CIN3 lesions and SCC; decreased PC coverage | Decreased median tumor volume | (Pietras et al., 2008) |
| Pancreatic islet tumor in RIP-Tag2 transgenic mouse model | Imatinib; CTX | PDGFR | N/A | No effect on tumor growth by imatinib; stopped tumor growth and reduced tumor burden in combination therapy | (Falcon et al., 2011) |
| Diffuse large B-cell lymphoma in human xenograft and EL4 lymphoma in murine allograft models | Imatinib | PDGFR, c-Kit, and BCR-Abl | Induced PC apoptosis and loss of perivascular integrity as well as microvascular density | Impaired lymphoma growth | (Ruan et al., 2013) |
| Melanoma in subcutaneous mouse model | Imatinib | PDGFR, c-Kit, and BCR-Abl | Inhibited vascular mimicry | Suppressed tumor growth | (Thijssen et al., 2018) |
| Pancreatic cancer in subcutaneous and orthotropic mouse models | TKI258 | PDGFR, VEGFR, FGFR | Impaired PC function and motility | Significant inhibition of tumor growth as well as reduced lymph node and liver metastases; delayed growth of established tumors and extended survival | (Taeger et al., 2011) |
| Ovarian carcinoma and NSCLC in subcutaneous mouse models | IMC-2C5^a^ | PDGFRβ | N/A | Decreased tumor growth rate in IMC-2C5 monotherapy in two mouse models | (Shen et al., 2009) |
| Lewis lung carcinoma in subcutaneous mouse model | CP673451 (PDGFRβ inhibitor) | PDGFRβ | Improved inhibition of PC was associated with increasing doses of CP673451 | CP673451 slowed the tumor growth without improving survival but enhanced metastasis in combined radiotherapy and Endostar therapy | (Yin et al., 2018) |
| Pancreatic cancer and CRC in subcutaneous and orthotropic mouse models | PDGF-BB (transfected); followed by imatinib mesylate | PDGFR, c-Kit, and BCR-Abl | Increased the number of PC in PDGF-BB–overexpressing clones; imatinib mesylate treatment decreased the number of PC | Reduced tumor mass in PDGF-BB–overexpressing clones; imatinib mesylate treatment reversed the growth inhibition of tumor | (McCarty et al., 2007) |
| HaCaT skin carcinogenesis mouse model | hPDGF-B and mVEGF-164 (transfected); followed by imatinib | PDGFR, c-Kit, and BCR-Abl | Combined hPDGF-B and mVEGF-164 expression results in mature functional tumor vasculatures and higher microvessel density; decreased tumor vasculatures and reduced association of αSMA^+^ PC with the BV following treatment with imatinib | Coexpression of mVEGF-164 and hPDGF-B shows no significant effect on tumor growth as compared with mVEGF-164 transfectant tumors, but improves tumor organization; treatment with imatinib reverses the above effect | (Lederle et al., 2010) |
| Breast cancer in orthotropic PDGFRβ-TK^b^ transgenic mouse model | N/A | PDGFRβ knockout | Reduced BV and increased permeability in PC-depleted tumors | PC depletion enhanced intra-tumoral hypoxia, decreased tumor growth, and increased lung metastasis in advanced-stage hypoxic tumors | (Keskin et al., 2015) |
| Human gastric cancer in orthotropic mouse model | Nilotinib; everolimus | PDGFR; mTOR | Decreased stromal reactivity and PC coverage in nilotinib treatment alone; decreased microvessel density in everolimus monotherapy | Tumor growth and metastasis are not inhibited in nilotinib treatment alone but significantly decreased stromal reactivity; both of the growth rate and stromal reaction were reduced in combination treatment with everolimus | (Onoyama et al., 2013) |
| Lewis lung carcinoma in colonization mouse model and subcutaneous melanoma in human CD248 knock-in mouse model | MORAb-004^c^ | CD248 | Impaired tumor microvasculature maturation and dysfunctional tumor microvasculature | Reduced primary tumor growth and inhibited tumor metastasis | (Rybinski et al., 2015) |
| RENCA in subcutaneous mouse model | DLK1-based vaccine | DLK1 | Enhanced vascular normalization | Inhibited tumor growth and increased frequencies of CD8+ T cell tumor infiltrating lymphocytes | (Chi Sabins et al., 2013) |
| RENCA and melanoma in subcutaneous mouse models | DLK1- and DLK2-based vaccines (lvDLK1 and lvDLK2) | DLK1 and DLK2 | Decreased vascular pruning and increased PC coverage when vaccinated with lvDLK1 or with combined lvDLK1 and lvDLK2 | Combined vaccination leads to improved antitumor benefits as compare with single vaccine treatment; enhanced antitumor efficacy when further combined with PD-L1 blockade | (Fabian et al., 2017) |
| Infantile hemangioma in subcutaneous mouse model | NOTCH3 Decoy (NOTCH3 inhibitor) | NOTCH3 | Decreased blood flow, vessel caliber, and αSMA^+^ perivascular cell coverage | Disrupted IH development | (Edwards et al., 2017) |
| *Indirect PC-targeted antitumor therapy* | | | | | |
| **Cancer type** | **Treatment** | **Targets** | **Impact on tumor PCs or vasculature** | **Impact on tumor growth** | **Reference** |
| Lewis lung carcinoma in subcutaneous mouse model | Anti-Olfml3 antibody | Olfml3 | Reduced angiogenesis and PC coverage of tumor vessels | Inhibited tumor growth | (Miljkovic-Licina et al., 2012) |
| Melanoma, Lewis lung carcinoma in subcutaneous mouse models | N/A | CCL2 knockout | No effect on PC coverage, blood vessel density and function | Reduced tumor cell growth | (Wong et al., 2020) |
| Orthotropic GBM xenograft model | Ibrutinib | BMX kinase | Disrupted glioma stem cell-derived PCs and blood-tumor barrier; increased vascular permeability | Improved chemotherapy efficacy resulting in retarded tumor growth and extended survival | (Zhou et al., 2017) |
| Melanoma, Lewis lung carcinoma, fibrosarcoma in subcutaneous mouse models | G6-31^d^ | PDGF-B knockout;  VEGF-A | Reduced PC abundance in *pdgfb^ret/ret^* mice | No improvement of VEGF-A inhibition in PC-deficient *pdgfb^ret/ret^* mice tumor | (Nisancioglu et al., 2010) |
| Lewis lung carcinoma mouse model and pancreatic islet tumor in RIP-Tag2 transgenic mouse | AX102^e^ | PDGF-B | Reduced PC and tumor vascularity | No decrease in tumor size in Lewis lung carcinoma; less prominent antitumor effect in pancreatic islet tumor. | (Sennino et al., 2007) |
| Lewis lung carcinoma mouse model and pancreatic islet tumor RIP-Tag2 mouse | AX102; CTX | PDGF-B | Decreased tumor vascularity in AX102 treatment in LLC tumors | Greater efficacy of CTX on tumors when combined with AX102 | (Falcon et al., 2011) |
| Highly metastatic human ovarian carcinoma in orthotropic mouse model | AX102; bevacizumab | PDGF-B; VEGF | Decreased PC coverage in the AX102 treatment group; reduced microvessel density in the bevacizumab treatment group | AX102 monotherapy is not effective; combination with bevacizumab treatment is highly efficacious | (Lu et al., 2010) |
| RCC, ovarian cancer and HCC subcutaneous mouse models; human melanoma xenograft | MEDI3617^f^; bevacizumab; paclitaxel | Ang-2; VEGF | Reduced vessel lumen area and decreased functional vessels | Inhibited tumor growth by blocking tumor angiogenesis; delayed tumor growth when combining with chemotherapy and bevacizumab | (Leow et al., 2012) |
| GBM multiforme in orthotropic mouse model | Trebananib; aflibercept | Ang-1/2; VEGF | Reduced vascular permeability and improved PC coverage (with depletion of tumor-associated macrophages and increased intratumoral T lymphocytes in combination therapy | Extended survival in combination therapy | (Scholz et al., 2016) |
| Human colon cancer in orthotropic mouse model | L1-7[N] (Ang-2 inhibitor);  mL4-3 (Ang-1 inhibitor) | Ang-2; Ang-1 | Reduced tumor vessels and enhanced vascular normalization in Ang-2 inhibitor treatment including increased PC coverage and reduced EC sprouting | Little effect on the tumor vascularity with Ang-1 inhibitor treatment; suppressed tumor growth in Ang-2 inhibitor monotherapy and combination therapy with Ang-1 inhibitor | (Falcon et al., 2009) |
| Lewis lung carcinoma, MT-ret melanoma, and B16F10 melanoma in Ang-2 knockout mice | N/A | Ang-2 knockout | Smaller microvessels, higher PC coverage and more mature PCs | Decreased tumor growth rate in all types of tumors in Ang-2 knockout mice | (Nasarre et al., 2009) |
| Breast cancer in orthotropic mouse model | Murinized  anti-ANG2 neutralizing antibody; imatinib | Ang-2; PDGFR | Anti-ANG2 antibody restores the integrity of PC-depleted leaky BV | Anti-ANG2 therapy significantly reduces the frequency of lung metastasis; combination of anti-ANG2 and imatinib treatment shows synergic effect on inhibiting primary tumor growth and lung metastasis as compared to imatinib monotherapy | (Keskin et al., 2015) |
| Melanoma lung metastatic experimental mice | TH10-DTX-NP^g^ | NG2-positive PC | Reduced PC density and microvessel density in the lung metastases | Extended tumor bearing mouse survival with no obvious toxicity and enhanced antitumor effect | (Guan et al., 2014) |
| Human NSCLC xenograft model | tTF-TAA^h^ | NG2-positive PC | Activated coagulation within the microvasculature | Suppressed tumor growth | (Brand et al., 2016) |
| Human breast cancer xenograft model | Z-GP-DAVLBH^i^ | FAPα-positive PC | Disrupted blood vessels in tumor core and periphery | Inhibited tumor growth or induced tumor regression | (Chen et al., 2017) |
| CRC in subcutaneous mouse model | Z-hTRAIL^j^ | PDGFRβ-positive PC | N/A | Increased tumor uptake and enhanced antitumor effect of hTRAIL contribute to tumor regression | (Tao et al., 2017) |
| BV: blood vessel; RENCA: murine renal carcinoma; CRC: colorectal cancer; CTX: cyclophosphamide; DLK: dual leucine zipper kinase; IH: infantile hemangioma; LLC, Lewis lung carcinoma; NSCLC: non-small-cell lung cancer; PDGF: platelet-derived growth factor; PDGFR: platelet-derived growth factor receptor; RCC: renal cell carcinoma; SCC: squamous cell carcinoma; VEGFR: vascular endothelial growth factor receptor | | | | | |

1. A fully human neutralizing antibody that directs against PDGFRβ;
2. Transgenic mice that express viral thymidine kinase under the PDGFRβ promoter as an alternative model for PC depletion;
3. An anti-human CD248 antibody Fb5;
4. A specific anti-VEGF-A antibody that neutralizes both murine and human VEGF-A;
5. A novel DNA oligonucleotide aptamer highly selective PDGF-B;
6. A human anti-angiopoietin 2 monoclonal antibody;
7. TH10 peptide conjugated nanoparticles loading docetaxel (TH10-DTX-NP) target the NG2 proteoglycan to facilitate nanoparticle internalization in PCs resulting in DTX-induced PC apoptosis;
8. tTF-TAA consisting of the extracellular domain of tissue factor (TF) and the peptides which represent ligands of NG2 target to PC leading to tumor vessel infarction;
9. Z-GP-DAVLBH hydrolyzed by FAPα to release vascular disrupting agent DAVLBH in order to disrupt the cytoskeleton of PC to overcome VDA treatment resistance;
10. Fused Z_PDGFR_ affibody mediates PC-targeted delivery of hTRAIL.

**References:**

Bergers, G., Song, S., Meyer-Morse, N., Bergsland, E., and Hanahan, D. (2003). Benefits of targeting both pericytes and endothelial cells in the tumor vasculature with kinase inhibitors. *J Clin Invest* 111(9)**,** 1287-1295. doi: 10.1172/JCI17929.

Brand, C., Schliemann, C., Ring, J., Kessler, T., Baumer, S., Angenendt, L., et al. (2016). NG2 proteoglycan as a pericyte target for anticancer therapy by tumor vessel infarction with retargeted tissue factor. *Oncotarget* 7(6)**,** 6774-6789. doi: 10.18632/oncotarget.6725.

Chen, M., Lei, X., Shi, C., Huang, M., Li, X., Wu, B., et al. (2017). Pericyte-targeting prodrug overcomes tumor resistance to vascular disrupting agents. *J Clin Invest* 127(10)**,** 3689-3701. doi: 10.1172/jci94258.

Chi Sabins, N., Taylor, J.L., Fabian, K.P., Appleman, L.J., Maranchie, J.K., Stolz, D.B., et al. (2013). DLK1: a novel target for immunotherapeutic remodeling of the tumor blood vasculature. *Mol Ther* 21(10)**,** 1958-1968. doi: 10.1038/mt.2013.133.

Edwards, A.K., Glithero, K., Grzesik, P., Kitajewski, A.A., Munabi, N.C., Hardy, K., et al. (2017). NOTCH3 regulates stem-to-mural cell differentiation in infantile hemangioma. *JCI Insight* 2(21). doi: 10.1172/jci.insight.93764.

Fabian, K.P.L., Chi-Sabins, N., Taylor, J.L., Fecek, R., Weinstein, A., and Storkus, W.J. (2017). Therapeutic efficacy of combined vaccination against tumor pericyte-associated antigens DLK1 and DLK2 in mice. *OncoImmunology* 6(3). doi: 10.1080/2162402X.2017.1290035.

Falcon, B.L., Hashizume, H., Koumoutsakos, P., Chou, J., Bready, J.V., Coxon, A., et al. (2009). Contrasting actions of selective inhibitors of angiopoietin-1 and angiopoietin-2 on the normalization of tumor blood vessels. *Am J Pathol* 175(5)**,** 2159-2170. doi: 10.2353/ajpath.2009.090391.

Falcon, B.L., Pietras, K., Chou, J., Chen, D., Sennino, B., Hanahan, D., et al. (2011). Increased vascular delivery and efficacy of chemotherapy after inhibition of platelet-derived growth factor-B. *Am J Pathol* 178(6)**,** 2920-2930. doi: 10.1016/j.ajpath.2011.02.019.

Guan, Y.Y., Luan, X., Xu, J.R., Liu, Y.R., Lu, Q., Wang, C., et al. (2014). Selective eradication of tumor vascular pericytes by peptide-conjugated nanoparticles for antiangiogenic therapy of melanoma lung metastasis. *Biomaterials* 35(9)**,** 3060-3070. doi: 10.1016/j.biomaterials.2013.12.027.

Keskin, D., Kim, J., Cooke, V.G., Wu, C.C., Sugimoto, H., Gu, C., et al. (2015). Targeting vascular pericytes in hypoxic tumors increases lung metastasis via angiopoietin-2. *Cell Rep* 10(7)**,** 1066-1081. doi: 10.1016/j.celrep.2015.01.035.

Kłosowska-Wardega, A., Hasumi, Y., Burmakin, M., Ahgren, A., Stuhr, L., Moen, I., et al. (2009). Combined anti-angiogenic therapy targeting PDGF and VEGF receptors lowers the interstitial fluid pressure in a murine experimental carcinoma. *PLoS One* 4(12)**,** e8149. doi: 10.1371/journal.pone.0008149.

Kuhnert, F., Tam, B.Y., Sennino, B., Gray, J.T., Yuan, J., Jocson, A., et al. (2008). Soluble receptor-mediated selective inhibition of VEGFR and PDGFRbeta signaling during physiologic and tumor angiogenesis. *Proc Natl Acad Sci U S A* 105(29)**,** 10185-10190. doi: 10.1073/pnas.0803194105.

Lederle, W., Linde, N., Heusel, J., Bzyl, J., Woenne, E.C., Zwick, S., et al. (2010). Platelet-derived growth factor-B normalizes micromorphology and vessel function in vascular endothelial growth factor-A-induced squamous cell carcinomas. *Am J Pathol* 176(2)**,** 981-994. doi: 10.2353/ajpath.2010.080998.

Leow, C.C., Coffman, K., Inigo, I., Breen, S., Czapiga, M., Soukharev, S., et al. (2012). MEDI3617, a human anti-angiopoietin 2 monoclonal antibody, inhibits angiogenesis and tumor growth in human tumor xenograft models. *Int J Oncol* 40(5)**,** 1321-1330. doi: 10.3892/ijo.2012.1366.

Lu, C., Kamat, A.A., Lin, Y.G., Merritt, W.M., Landen, C.N., Kim, T.J., et al. (2007). Dual targeting of endothelial cells and pericytes in antivascular therapy for ovarian carcinoma. *Clin Cancer Res* 13(14)**,** 4209-4217. doi: 10.1158/1078-0432.CCR-07-0197.

Lu, C., Shahzad, M.M., Moreno-Smith, M., Lin, Y.G., Jennings, N.B., Allen, J.K., et al. (2010). Targeting pericytes with a PDGF-B aptamer in human ovarian carcinoma models. *Cancer Biol Ther* 9(3)**,** 176-182. doi: 10.4161/cbt.9.3.10635.

McCarty, M.F., Somcio, R.J., Stoeltzing, O., Wey, J., Fan, F., Liu, W., et al. (2007). Overexpression of PDGF-BB decreases colorectal and pancreatic cancer growth by increasing tumor pericyte content. *Journal of Clinical Investigation* 117(8)**,** 2114-2122. doi: 10.1172/JCI31334.

Miljkovic-Licina, M., Hammel, P., Garrido-Urbani, S., Lee, B.P., Meguenani, M., Chaabane, C., et al. (2012). Targeting olfactomedin-like 3 inhibits tumor growth by impairing angiogenesis and pericyte coverage. *Mol Cancer Ther* 11(12)**,** 2588-2599. doi: 10.1158/1535-7163.MCT-12-0245.

Nasarre, P., Thomas, M., Kruse, K., Helfrich, I., Wolter, V., Deppermann, C., et al. (2009). Host-derived angiopoietin-2 affects early stages of tumor development and vessel maturation but is dispensable for later stages of tumor growth. *Cancer Res* 69(4)**,** 1324-1333. doi: 10.1158/0008-5472.CAN-08-3030.

Nisancioglu, M.H., Betsholtz, C., and Genove, G. (2010). The absence of pericytes does not increase the sensitivity of tumor vasculature to vascular endothelial growth factor-A blockade. *Cancer Res* 70(12)**,** 5109-5115. doi: 10.1158/0008-5472.CAN-09-4245.

Onoyama, M., Kitadai, Y., Tanaka, Y., Yuge, R., Shinagawa, K., Tanaka, S., et al. (2013). Combining molecular targeted drugs to inhibit both cancer cells and activated stromal cells in gastric cancer. *Neoplasia* 15(12)**,** 1391-1399. doi: 10.1593/neo.131668.

Pietras, K., and Hanahan, D. (2005). A multitargeted, metronomic, and maximum-tolerated dose "chemo-switch" regimen is antiangiogenic, producing objective responses and survival benefit in a mouse model of cancer. *J Clin Oncol* 23(5)**,** 939-952. doi: 10.1200/jco.2005.07.093.

Pietras, K., Pahler, J., Bergers, G., and Hanahan, D. (2008). Functions of paracrine PDGF signaling in the proangiogenic tumor stroma revealed by pharmacological targeting. *PLoS Med* 5(1)**,** e19. doi: 10.1371/journal.pmed.0050019.

Ruan, J., Luo, M., Wang, C., Fan, L., Yang, S.N., Cardenas, M., et al. (2013). Imatinib disrupts lymphoma angiogenesis by targeting vascular pericytes. *Blood* 121(6)**,** 5192-5202. doi: 10.1182/blood-2013-03-490763.

Rybinski, K., Imtiyaz, H.Z., Mittica, B., Drozdowski, B., Fulmer, J., Furuuchi, K., et al. (2015). Targeting endosialin/CD248 through antibody-mediated internalization results in impaired pericyte maturation and dysfunctional tumor microvasculature. *Oncotarget* 6(28)**,** 25429-25440. doi: 10.18632/oncotarget.4559.

Schiffmann, L.M., Brunold, M., Liwschitz, M., Goede, V., Loges, S., Wroblewski, M., et al. (2017). A combination of low-dose bevacizumab and imatinib enhances vascular normalisation without inducing extracellular matrix deposition. *Br J Cancer* 116(5)**,** 600-608. doi: 10.1038/bjc.2017.13.

Scholz, A., Harter, P.N., Cremer, S., Yalcin, B.H., Gurnik, S., Yamaji, M., et al. (2016). Endothelial cell-derived angiopoietin-2 is a therapeutic target in treatment-naive and bevacizumab-resistant glioblastoma. *EMBO Mol Med* 8(1)**,** 39-57. doi: 10.15252/emmm.201505505.

Sennino, B., Falcon, B.L., McCauley, D., Le, T., McCauley, T., Kurz, J.C., et al. (2007). Sequential loss of tumor vessel pericytes and endothelial cells after inhibition of platelet-derived growth factor B by selective aptamer AX102. *Cancer Res* 67(15)**,** 7358-7367. doi: 10.1158/0008-5472.CAN-07-0293.

Shen, J., Vil, M.D., Prewett, M., Damoci, C., Zhang, H., Li, H., et al. (2009). Development of a fully human anti-PDGFRbeta antibody that suppresses growth of human tumor xenografts and enhances antitumor activity of an anti-VEGFR2 antibody. *Neoplasia* 11(6)**,** 594-604. doi: 10.1593/neo.09278.

Taeger, J., Moser, C., Hellerbrand, C., Mycielska, M.E., Glockzin, G., Schlitt, H.J., et al. (2011). Targeting FGFR/PDGFR/VEGFR impairs tumor growth, angiogenesis, and metastasis by effects on tumor cells, endothelial cells, and pericytes in pancreatic cancer. *Mol Cancer Ther* 10(11)**,** 2157-2167. doi: 10.1158/1535-7163.MCT-11-0312.

Tao, Z., Yang, H., Shi, Q., Fan, Q., Wan, L., and Lu, X. (2017). Targeted Delivery to Tumor-associated Pericytes via an Affibody with High Affinity for PDGFRβ Enhances the in vivo Antitumor Effects of Human TRAIL. *Theranostics* 7(8)**,** 2261-2276. doi: 10.7150/thno.19091.

Thijssen, V.L.J.L., Paulis, Y.W.J., Nowak-Sliwinska, P., Deumelandt, K.L., Hosaka, K., Soetekouw, P.M.M.B., et al. (2018). Targeting PDGF-mediated recruitment of pericytes blocks vascular mimicry and tumor growth. *Journal of Pathology* 246(4)**,** 447-458. doi: 10.1002/path.5152.

Wong, P.P., Munoz-Felix, J.M., Hijazi, M., Kim, H., Robinson, S.D., De Luxan-Delgado, B., et al. (2020). Cancer Burden Is Controlled by Mural Cell-beta3-Integrin Regulated Crosstalk with Tumor Cells. *Cell* 181(6)**,** 1346-1363 e1321. doi: 10.1016/j.cell.2020.02.003.

Yin, L., He, J., Xue, J., Na, F., Tong, R., Wang, J., et al. (2018). PDGFR-beta inhibitor slows tumor growth but increases metastasis in combined radiotherapy and Endostar therapy. *Biomed Pharmacother* 99**,** 615-621. doi: 10.1016/j.biopha.2018.01.095.

Zhou, W., Chen, C., Shi, Y., Wu, Q., Gimple, R.C., Fang, X., et al. (2017). Targeting Glioma Stem Cell-Derived Pericytes Disrupts the Blood-Tumor Barrier and Improves Chemotherapeutic Efficacy. *Cell Stem Cell* 21(5)**,** 591-603.e594. doi: 10.1016/j.stem.2017.10.002.
